# Effect of Camelina and Linseed Cake Supplementation on the Antioxidant and Amino Acid Contents, Oxidative Stability, Water Activity and Sensory Attributes of *Tenebrio molitor* Larvae

**DOI:** 10.3390/foods15040787

**Published:** 2026-02-22

**Authors:** Antonella Dalle Zotte, Zdeněk Volek, Marco Cullere, Emanuele Pontalti, Bianca Palumbo

**Affiliations:** 1 Department of Animal Medicine, Production and Health, University of Padova, Agripolis, Viale dell’Università 16, Legnaro, 35020 Padova, Italy; marco.cullere@unipd.it (M.C.); emanuele.pontalti@phd.unipd.it (E.P.); bianca.palumbo@unipd.it (B.P.); 2 Institute of Animal Science, Přátelství 815, 104 00 Prague, Czech Republic; 3 Department of Microbiology, Nutrition and Dietetics, Faculty of Agrobiology, Food and Natural Sciences, Czech University of Life Sciences Prague, Kamýcká 129, Suchdol, 165 00 Prague, Czech Republic

**Keywords:** agro-by-products, yellow mealworm, lipid stability, omega-3, sensory evaluation

## Abstract

Camelina and linseed cakes were included in the diet of *Tenebrio molitor* (TM) larvae at two levels (5% and 10%) to evaluate their effects on antioxidant and amino acid contents, oxidative stability, water activity (a_w_), and sensory attributes. Six experimental diets were tested: a standard diet used by the insect farm (STD), a commercial control diet (CON), and CON with two inclusion levels of camelina (CAM 5, CAM 10) or linseed (LIN 5, LIN 10) cakes. Each treatment consisted of 12 replicates of five-week-old larvae reared until commercial size (9 weeks). Camelina and linseed cake inclusion affected the a_w_ of dried larvae, with the highest values in CAM 5 and the lowest in LIN 10 (0.69 vs. 0.45, respectively; *p* = 0.016). The highest linseed inclusion level increased susceptibility to lipid oxidation during storage (11.3 vs. an average 2.93 meq O_2_/kg fat, respectively; *p* < 0.0001), despite elevated antioxidant concentrations (α, δ, γ -tocopherols and β-carotene). Larvae fed with CAM 5 and LIN 5 diets had a higher content of most essential amino acids compared to the other treatments (*p* < 0.0001). Conversely, increasing the inclusion level to 10% determined a reduction in total amino acid content and in key essential amino acids, particularly lysine (*p* < 0.0001). Non-essential amino acids displayed a similar trend, except glycine, whose highest value was observed in the LIN 10 group (933 vs. 652 mg/100 g, on average). Sensory evaluation showed that LIN 10 larvae achieved the highest scores for visual and overall acceptability, although some results need further investigation. Overall, camelina and linseed cakes appear to be promising, sustainable agro-industrial by-products to be exploited in TM farming, especially at moderate inclusion levels, as the nutritional quality and market appeal of TM biomass were ensured.

## 1. Introduction

In recent years, insects have been proposed as an alternative and sustainable source of feed and food in response to the global challenges that are expected to arise in the near future [1]. Among edible insects, *Tenebrio molitor* (TM) is one of the most extensively studied species in Europe, also due to its rich nutritional profile and lower environmental impact compared with conventional livestock. In particular, TM larvae are characterized by a high protein content (approximately 50% of dry matter—DM), providing most essential amino acids, and by a lipid fraction of around 30% (DM), which is rich in polyunsaturated fatty acids (PUFA), notably oleic, linoleic and α-linolenic acids [2,3,4]. However, the chemical composition of TM larvae is strongly influenced by the feeding substrate, which is commonly supplemented with additional sources of protein, vitamins, and minerals to enhance productivity, shorten development time, and improve growth and survival [5,6,7]. Additionally, the feeding substrate plays a key role in shaping the chemical composition of TM larvae; in particular, the fatty acid (FA) profile largely reflects that of the diet [8,9,10]. Consequently, substrate selection is a crucial factor in TM rearing, as dietary manipulation may influence both nutritional quality and potential applications of the larvae as food and feed ingredients. TM larvae are generally characterized by a high PUFA content, with a predominance of *n*-6 FA and relatively low *n*-3 FA levels, resulting in an unbalanced *n*-6/*n*-3 ratio [2,10,11]. This feature may limit the broader use of TM larvae as food and feed ingredients, as an unbalanced *n*-6/*n*-3 ratio is widely recognized as a critical aspect of nutritional quality in both human and animal diets [12,13,14,15,16,17]. This aspect has prompted growing interest in dietary strategies aimed at modulating larval lipid composition.

In this context, including *n-3*-rich ingredients in the diet of TM larvae represents a promising approach to improve their nutritional and technological quality. The inclusion of oilseed-derived ingredients in insect diets is of particular interest in the context of circular economy, as it allows the valorization of agro-industrial by-products while providing nutritionally valuable feed components. In the present study, camelina and linseed cakes, by-products of oil extraction from *Camelina sativa* L. Crantz and *Linum usitatissimum* L., respectively, were included in the diet of TM larvae with the aim of improving larval quality and assessing the effects of dietary inclusion on oxidative stability and sensory acceptability. These oilseed cakes are rich in protein (approximately 35% of DM), residual oil (7–22% of DM, depending on the extraction process), and bioactive compounds, including natural antioxidants [18,19,20]. However, the inclusion of PUFA-rich ingredients may increase the susceptibility of insect-derived products to oxidative processes, potentially leading to quality deterioration and undesirable sensory attributes [21].

Moreover, antinutritional factors present in these by-products—such as cyanogenic glycosides, phytic acid, and tannins in linseed cake, and glucosinolates, phytic acid, erucic acid, and sinapine in camelina cake—which may negatively affect insect performance if inappropriate inclusion levels are used [22,23,24,25]. Therefore, identifying suitable feeding strategies and inclusion levels that enhance larval quality while preserving oxidative stability, sensory properties, and minimizing the negative effects of antinutritional factors remains a critical challenge. While the effects of oilseed cakes on the lipid profile of insects appear to be relatively direct, their impact on protein deposition and amino acid content remains largely unexplored, yet pivotal in order to understand the full potential of the innovative feedstuff and to find the optimum inclusion levels ensuring maximum larvae growth and product nutritional quality.

Despite growing interest in the use of oilseed cakes in insect rearing, limited information is available on their effects on oxidative stability, antioxidant content, amino acid content and sensory properties of TM larvae. Addressing these aspects is essential to ensure an adequate formulation of diets, larvae nutritional quality, as well as the technological suitability and consumer acceptability of insect-based products. Therefore, the aim of the present study was to evaluate the effects of including camelina and linseed cakes at two dietary levels (5% and 10%) on the antioxidant and amino acids content, oxidative stability, water activity (a_w_) and sensory attributes of TM larvae. The preliminary results concerning the FA profile of diets and larvae were published in conference proceedings [26] and will therefore not be included in the present manuscript.

## 2. Materials and Methods

### 2.1. Experimental Design and Experimental Conditions

The experiment was conducted at the Insect Novel Ecologic Food (INEF) insects farm (Piombino Dese, Padova, Italy) from January to March 2025. The experiment utilized 5-week-old TM larvae that were collected and separated from frass and remaining feed material by sieving. To determine average individual larval mass, a subsample of 500 specimens was both counted and weighed, yielding a mean value of 7.68 ± 0.03 mg. TM larvae were randomly assigned to six dietary treatments: a diet routinely used at the INEF farm (STD), which served as a commercial control; a control formulation based on wheat bran, corn gluten meal, corn meal, full-fat soybean, soybean meal, and calcium carbonate (CON); the same control diet formulated to include: 5% camelina cake (CAM 5), 10% camelina cake (CAM 10), 5% linseed cake (LIN 5) and 10% linseed cake (LIN 10). All diets, except the STD diet, were formulated to be isonitrogenous (crude protein: 171, 177, 176, 175, 178 and 181 g/kg as is basis for STD, CON, CAM 5, CAM 10, LIN 5, and LIN 10, respectively) and isoenergy (gross energy: 16.3, 17.4, 17.2, 17.5, 17.4, and 17.9 MJ/kg as is basis for STD, CON, CAM 5, CAM 10, LIN 5, and LIN 10, respectively), and each treatment included 12 replicates. Larvae were reared in plastic crates (60 × 40 × 14.5 cm, surface area = 2400 cm^2^) at a density of 4.2 larvae/cm^2^, as commonly applied at the insect farm. Each crate received 76.8 g of larvae, calculated from the average weight of 5-week-old individuals. The crates were then supplied with ground feed of uniform particle size matching their assigned treatment, allowing for *ad libitum* consumption for the duration of the experiment (4 weeks, until 9 weeks of age when larvae reached the commercial selling size).

The rearing facility maintained controlled environmental parameters with a mean temperature of 28.0 ± 2.3 °C and a relative humidity of 70.1 ± 10%. A constant photoperiod regime of 8:16 h (light: dark) was implemented throughout the study. Continuous monitoring and automated regulation of all environmental conditions were achieved through facility equipment, including heating/cooling units and a humidification system.

### 2.2. Shelf Life of Larvae and Water Activity

A subsample of larvae of 9 weeks of age, from each crate and within each experimental group, was microwave-dried using a Max Industrial Microwave 30B (Max Industrial Microwave, Yantai, Shandong, China) at 103 °C (12 kW) for 10 min. This drying method, routinely applied at the INEF insect farm, was employed to obtain standardized material for both sensory evaluation and shelf-life assessment. The a_w_ of dried larvae was measured using an AquaLab CX-2 instrument (Meter Group, Munich, Germany), with measurements performed in duplicate, following the operational indications. All the technical specification of the instrument are presented in the Operator’s Manual version 3.0: https://library.metergroup.com/Retired%20and%20Discontinued/Manuals/AquaLabCX-2v3.pdf (accessed on 17 March 2025). Shelf-life evaluation included the quantification of antioxidant compounds (β-carotene, α-tocopherol, δ-tocopherol, and γ-tocopherol) and the determination of peroxide value. Analyses were performed immediately after processing (T0) and after 3 months of storage at room temperature in plastic closed containers (T3).

### 2.3. Chemical Analyses

At the end of the trial, a sample of larvae (500 g) from each crate was collected for further analysis. The peroxide value of microwave dried larvae was assessed according to AOCS official method Cd 8-53 [27]. This analysis was performed both at the end of the experiment (T0) and after 3 months of storage of larvae (T3).

The contents of ß-carotene, α-tocopherol, δ-tocopherol, and γ-tocopherol in diets, cakes and larvae were determined following the European standards [28,29] by high-performance liquid chromatography, equipped with a diode-array detector (VP series) (Shimadzu, Kyoto, Japan). Chromatographic analyses were performed by means of a Phenomenex Synergy 4 mm Fusion-RP 80 Å column (150 × 4.6 mm, 4 mm; P/No. 00F-4424-E0, Torrance, CA, USA); methanol was the mobile phase. The method had a gradient flow program, where the solvent composition remained constant and the flow rate was adjusted from 0.6 mL/min to 1.5 mL/min and then back to 0.6 mL/min. The sample injection volume was 50 mL. For each compound, quantification was performed using individual calibration curves. Detection wavelengths were 292 nm for α-tocopherol, 296 nm for δ and γ tocopherols, and 450 nm for β-carotene. Antioxidants levels of diets, camelina and linseed cakes are reported in Table 1.

The amino acids content of the cakes, diets (Table 2) and of TM larvae was determined after a pretreatment with acid hydrolysis by amino analyzer at the Department of Animal Medicine, Production and Health (MAPS). In detail, 500 mg of sample were placed into a glass vial in which 3.75 mL of a 2.5% solution of 3,3-dithiopropionic acid prepared in 0.2 M sodium hydroxide (NaOH) was added. After subsequent addition of 3.75 mL of hydrochloric acid (37% HCl), the vial was mixed thoroughly to ensure sample homogeneity and placed it in an oven for 24 h (105 °C). During this hydrolysis period, each sample was gently mixed every hour. Afterwards, the entire content of the vial was transferred into a round-bottom flask (100 mL) and diluted using distilled water to a total volume of 100 mL. After mixing, 5 mL of the diluted hydrolysate were filtered through a 0.45 μm syringe filter into a clean vial. For the subsequent chromatographic analysis, 100 μL of the filtered solution were added to 900 μL of Mobile Phase A (AA-MA(Li)). Samples were then injected in a Shimadzu High Performance Liquid Chromatograph (Nexera Amino Acid Analysis System); after post column derivatization, the sample had a column flow (Li-type Analysis Mode with Shim-pack Amino-Li column) rate 0.6 mL/min and a gradient program of mobile phase was applied. The column oven temperature was 39 °C, injection volume was 20 µL and the amino acids determination was determined with Shimadzu Fluorescence Detection RF-20A XS (excitation wavelength: 350 nm; fluorescence wavelength: 450 nm). The amino acids amounts were obtained using a 5-point calibration curve.

### 2.4. Sensory Analysis of Larvae

Larvae were subjected to two distinct sensory evaluations: a consumer sensory analysis and an objective sensory evaluation by a trained panel.

For the consumer sensory analysis, 141 volunteers comprising employees and students from the University of Padova (Italy), aged 20–70 years, participated in the study. As a preference test was employed, prior experience with the specific food matrix or sensory evaluation methods was not required. Each participant received a detailed guide outlining the correct evaluation procedure. Subsequently, participants assessed six samples of dried TM larvae, corresponding to different treatments and labeled A, B, C, D, E, and F, based on visual, olfactory, and overall acceptability. Each attribute was scored on a 7-point scale, ranging from 1 (extremely unacceptable) to 7 (extremely acceptable). In the present experiment, a score > 4 represented a positive finding since the product tends to the acceptability target. The sensory evaluation excluded tasting. Prior to the test, TM larvae underwent microbiological analysis for common pathogens to ensure product safety. However, participants were not permitted to touch the samples.

For the objective sensory evaluation, six samples (one per treatment) of dried TM larvae were coded with random three-digit numbers. A total of 22 trained panelists, aged 25–65 years, participated in the test, which consisted of a descriptive sensory analysis. They underwent a 2 h pre-test training session to become familiar with the matrices and to select appropriate descriptors. In addition, a list of possible descriptors and off-flavors was prepared. Dried larvae used for the training session were obtained from insects reared on a conventional diet at the INEF farm; they were handled and dried in the same way as the samples used for the subsequent sensory analysis. All panelists signed consent forms and provided informed consent prior to the sensory evaluation.

For the evaluation, panelists received a list of descriptors to be scored on continuous 15 cm scales, ranging from 1 (lowest intensity) to 9 (highest intensity). The selected descriptors included: color intensity, size uniformity, general odor intensity, rancid odor intensity, fried odor intensity, peanut odor intensity, unctuosity of whole larvae, unctuosity of minced larvae, and friability. All the evaluations were performed in a room where the temperature was set at 22 °C. Panelists were instructed to sniff, observe, and touch the samples, but not to taste them. Scores were recorded by measuring the position on the 15 cm scale, and data were expressed in millimeters for statistical analysis.

### 2.5. Statistical Analysis

Data were preliminarily tested for normality by using the Shapiro–Wilk test: values <0.9 were considered as normal. Experimental data were analyzed following the GLM procedures of the SAS 9.1.3 statistical analysis software for Windows [30]. A one-way ANOVA tested the effects of diet and time (fixed effects) on the peroxide values and antioxidants of dried TM larvae. Another one-way ANOVA tested the effects of diet (fixed effect) on the amino acids content and a_w_ of dried TM larvae. Two distinct mixed models (PROC MIXED) were used to analyze consumers’ and panel sensory analysis data: in both cases, diet was considered as fixed effect, while consumers/panelists were included as random effect. Least square means were obtained using a Bonferroni test, and the significance was calculated at a 5% confidence level.

## 3. Results

### 3.1. Peroxide Value, Antioxidant and Amino Acids Contents, and Water Activity of Dried Larvae

Results pertaining to the effect of camelina and linseed cake dietary inclusion on the peroxide value (meq O_2_/kg of fat) of dried TM larvae, evaluated at time 0 (T0) and after 3 months of storage (T3) are presented in Table 3. No significant differences in lipid oxidation were observed among the six experimental groups at T0. However, a significant effect emerged at T3: larvae from the LIN 10 group showed a higher peroxide value compared with the other groups (11.3 vs. 2.93 meq O_2_/kg fat, respectively; *p* < 0.0001). Moreover, only in the LIN 10 group, the peroxide value increased significantly over the 3-month storage period, while no changes were detected in the other groups (11.3 vs. 2.83 meq O_2_/kg fat; *p* < 0.0001).

Results concerning the effects of a dietary inclusion of camelina and linseed cake on α-tocopherol, δ-tocopherol, γ-tocopherol and β-carotene (mg/kg of larvae) of dried TM larvae, evaluated at time 0 (T0) and after 3 months of storage (T3), are presented in Table 4. Regarding the antioxidant levels of larvae at T0, the α-tocopherol content was the highest in the CAM 10 (7.17 mg/kg) and the lowest in the CON (3.79 mg/kg; *p* < 0.0001) groups. At T3, CAM 5 showed the highest value (6.81 mg/kg), whereas LIN 5 recorded the lowest (4.03 mg/kg). Overall, no significant differences were observed between T0 and T3 within each group.

For δ-tocopherol, all groups supplemented with oilseed cakes showed markedly higher levels than STD (0.23 mg/kg) at T0 (*p* < 0.0001). The highest concentrations were observed in LIN 10 and CAM 10 (5.44 mg/kg and 5.27 mg/kg). A similar trend was found at T3, with LIN 10 and CAM 10 maintaining the highest levels and STD diet the lowest (4.81 mg/kg vs 0.16 mg/kg, on average, respectively; *p* < 0.0001).

The amount of γ-tocopherol was strongly influenced by diet. At T0, STD larvae showed the lowest content (2.11 mg/kg), while the highest levels were recorded in LIN 10 and CAM 10 (10.5 mg/kg, on average; *p* < 0.0001). At T3, values decreased in all groups except CAM 5, although CAM 10 still showed the highest level and STD the lowest (10.6 vs. 1.46 mg/kg, respectively; *p* < 0.0001).

For β-carotene, STD and LIN 10 larvae displayed the highest initial contents (1.40 and 1.53 mg/kg, respectively), whereas CON showed the lowest (0.81 mg/kg; *p* < 0.0001). At T3, concentrations remained stable, with LIN 10 exhibiting the highest value and LIN 5 the lowest (1.28 vs. 0.79 mg/kg; *p* = 0.0011).

Dietary inclusion of camelina and linseed cakes significantly affected both essential and non-essential amino acid contents of TM larvae (Table 5). Larvae fed CAM 5 and LIN 5 diets showed higher contents of several essential amino acids such as histidine, isoleucine, threonine and valine, compared with the other treatments (*p* < 0.001). Conversely, increasing the inclusion level to 10% resulted in a reduction in total amino acid content and of key essential amino acids, particularly lysine (*p* < 0.001). Non-essential amino acids, including proline, serine and tyrosine, followed a similar trend, with higher concentrations at 5% inclusion compared with both CON and standard STD diets, and reduced values at 10%. Glycine showed an opposite trend, with the highest value observed in the LIN 10 group (933 vs. 652 mg/100 g fresh weight, on average). Camelina and linseed cakes at 5% inclusion led to the highest total amino acid content in larvae (13,294 mg/100 g and 13,881 mg/100 g fresh weight, respectively).

**Figure 1:**
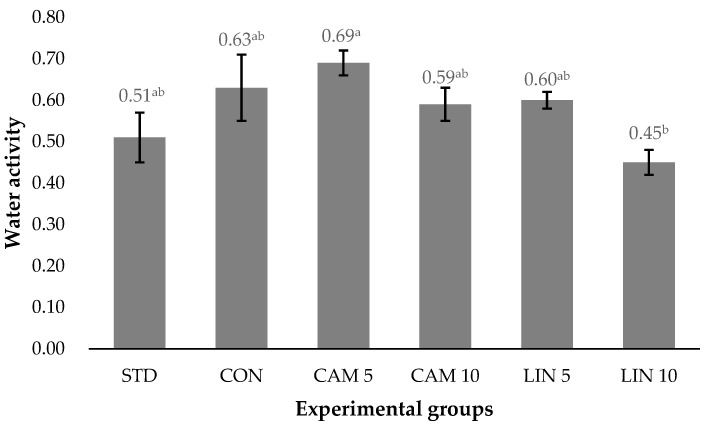
Effect of camelina and linseed cake dietary inclusion on water activity of 9 weeks old *Tenebrio molitor* larvae. STD: diet commonly used by the farm; CON: standard diet; CAM 5: standard diet supplemented with 5% of camelina cake; CAM 10: standard diet supplemented with 10% of camelina cake; LIN 5: standard diet supplemented with 5% of linseed cake; LIN 10: standard diet supplemented with 10% of linseed cake; ^a,b^ values within a row with different superscripts differ significantly at *p* < 0.05.

The results of a_w_ of dried larvae are shown in Figure 1. Significant differences in a_w_ were observed among groups, despite the same drying method being applied. In particular, larvae from the CAM 5 group exhibited the highest a_w_ value, whereas those from the LIN 10 group showed the lowest (0.69 vs. 0.45, respectively; *p* = 0.016). The remaining groups displayed intermediate values, averaging 0.58.

### 3.2. Sensory Evaluation of Dried Larvae

Results of the consumer sensory analysis are presented in Table 6. Larvae of LIN 10 group received the highest score for visual acceptance, followed by CAM 10, whereas CON scored the lowest (5.05 and 4.89 vs. 4.10, respectively; *p* = 0.0032). LIN 10 also achieved the highest score for olfactory acceptance, together with STD, while CON again received the lowest (5.67 vs. 3.76, on average; *p* < 0.0001). The remaining groups showed intermediate values. A similar trend was observed for overall acceptance, with LIN 10 scoring the highest and CON the lowest (5.42 vs. 3.90, respectively; *p* < 0.0001).

Results of the panel sensory analysis are reported in Table 7. Color intensity was highest in the CAM 5 (85.9) group, while STD larvae showed the lowest value (61.5; *p* = 0.0032). No significant differences were detected in size uniformity among groups (*p* = 0.8158). Odor intensity was significantly affected by diet (*p* = 0.0002): LIN 10 larvae received the highest score (97.8), whereas LIN 5 recorded the lowest (67.7).

Rancid odor intensity did not differ significantly among groups (*p* = 0.2536). Conversely, fried odor intensity varied, with CAM 5, CAM 10 and LIN 10 larvae reporting the highest values and LIN 5 the lowest (57.2 vs. 39.0, on average respectively; *p* = 0.0028). Peanut odor intensity was also influenced by diet (*p* = 0.0001): LIN 10 and STD larvae obtained a higher score compared to the other groups (70.5 vs. 48.6, on average, respectively; *p* = 0.0001). Concerning unctuousness, whole STD larvae were judged less unctuous compared with oilseed cakes treated groups, while the CON group showed an intermediate value (22.1 vs. 38.9 vs. 34.4, on average, respectively; *p* = 0.0255). Also, fragmented larvae showed significant differences (*p* = 0.0383), with CAM 5 receiving the highest values, and STD the lowest (64.8 vs. 44.4, respectively; *p* = 0.038). Finally, friability was strongly affected by diet (*p* < 0.0001): LIN 10 and STD groups obtained the highest scores (109 and 112 respectively), while LIN 5, CON and CAM 5 showed the lowest (55.0).

## 4. Discussion

At T3, LIN 10 larvae showed a higher peroxide value compared with the other groups and the peroxide value increased significantly over the 3-month storage period in the LIN 10 group only. Differences among dietary treatments were also observed regarding the antioxidant contents at both T0 and T3. Variations in oxidative stability and tocopherol levels further emphasize the role of dietary lipid sources in determining product quality during storage. In the present study, the peroxide value was used to assess the extent of lipid oxidation in dried larvae. This parameter is a widely recognized indicator of the lipid oxidation degree, reflecting the concentration of primary oxidation products in the sample [31]. It was specifically measured to evaluate whether the inclusion of camelina and linseed cakes, both rich in highly unsaturated fatty acids, could increase the susceptibility of TM larvae lipids to oxidative degradation.

No specific guidelines are currently available regarding the quality of insect fat/oil; however, the European Food Safety Authority (EFSA), in its scientific opinion on frozen and dried whole TM larvae, reported that peroxide values of ≤5 meq O_2_/kg are considered acceptable for dried and powdered larvae [32]. In the present study, the peroxide values of all groups fell under the limit at both T0 and T3, except for the LIN 10 group. In this group the peroxide value increased significantly compared with T0 and was the highest among all experimental groups, highlighting a higher susceptibility to lipid oxidation after three months of storage. In fact, the final value (11.3 meq O_2_/kg of fat) resulted in being slightly above the recommended level of 10 meq/kg fat, the threshold above which oils are regarded as unstable and easily become rancid [33]. Since the same drying method was applied to all samples, the increased susceptibility of the LIN 10 group can be attributed to the specific composition of the larvae, resulting from the inclusion of 10% linseed cake in the diet. Notably, this group exhibited the highest concentrations of δ-tocopherol (together with CAM 10) at both T0 and T3, the highest γ-tocopherol level (along with CAM 10) at T0, and the highest β-carotene content. This is consistent with the composition of linseed and camelina cakes, which are particularly rich in γ-tocopherol (Table 1). Nevertheless, the higher peroxide value observed in the LIN 10 group at T3, despite its elevated levels of tocopherols and carotenoids, can be explained by the FA profile of the larvae. Linseed cake is rich in α-linolenic acid, which is highly prone to oxidation. Its inclusion in the diet markedly increased the α-linolenic acid content of LIN 10 larvae [26], reaching higher levels than in the other groups. Consequently, the abundance of oxidizable substrates in LIN 10 larvae likely exceeded the protective capacity of antioxidants, resulting in the observed higher peroxide value. This suggests that the balance between lipid composition and antioxidant content is critical, and that elevated antioxidant levels do not necessarily guarantee oxidative stability when the unsaturation degree is also high. Furthermore, it cannot be excluded that a portion of the tocopherols might have been modified or oxidized while protecting feed/food oxidation during storage, thereby reducing their effectiveness in counteracting oxidation.

In the present research it was observed that the dietary inclusion of camelina and linseed cakes influenced essential and non-essential amino acid contents of TM larvae. Research conducted up to now provides controversial indications regarding the effects of amino acid composition of the diet on TM larvae. One research study testing different agricultural by-products as rearing substrate for TM larvae observed that the amino acid profile of the larvae remained overall consistent despite changes in the rearing substrate. However, the overall nutritional composition of the larvae can be altered by the diet provided, therefore indicating some degree of homeostasis in larvae amino acid composition regardless of diet variations [34]. Further research, instead, showed that different protein sources, and more specifically the quality of feed proteins (amino acids), could play a significant role in the mealworm larvae protein and amino acid contents [35,36]. The fact that TM larvae can modulate their amino acid profile in response to dietary composition was also highlighted by [37], where some essential amino acids like valine, arginine, and leucine varied according to the rearing substrate.

In the present research, the amino acid composition of the rearing substrate was also found to be pivotal in shaping the amino acid content of larvae, thus further emphasizing that TM larvae modulate their amino acid profile and content in response to dietary provision. In addition, it was observed that with LIN 5 and CAM 5 (moderate inclusion levels) the amino acid deposition was enhanced, whereas higher inclusion levels (10%) seemed to impair protein utilization. This could be explained by the fact that high amounts of dietary amino acids (surplus amino acids) are typically poured out in excrements by TM larvae, which is linked to the anatomical structure of the insect species for which no significant amounts of amino acids are required for the construction of muscle mass [37]. Additionally, a complementary hypothesis to explain the present findings could be that with higher cakes’ inclusion levels the dietary content of antinutritional factors also increases, thus worsening the digestibility of nutrients. In fact, camelina and linseed contain glucosinolates, phytic acid, and tannins while camelina also contains erucic acid [25,38]. However, the possible impact of such antinutritional factors on TM larvae digestibility and nutrients’ absorption should be carefully investigated as no adequate research has been conducted up to now.

Despite their increased susceptibility to lipid oxidation, larvae from the LIN 10 group received favorable sensory scores, indicating positive consumer perception. Results of the present study demonstrate that, even though the larvae were not tasted, consumers perceived clear differences in their appearance and olfactory attributes. The inclusion of oilseed cakes enriched the larvae in PUFAs [26] which are likely to influence visual and olfactory attributes [39,40]. As a result, LIN 10 larvae obtained the highest overall acceptance scores in consumer tests. More specifically, they achieved the best score for visual acceptability, followed by the CAM 10 group. In terms of olfactory acceptability, however, the LIN 10 larvae shared the top score with the STD group. The sensory panel analysis further confirmed that dietary treatments affected the sensory attributes of larvae. However, some findings from both consumers and panel require further investigation, as certain patterns remain unclear. For instance, differences emerged among the groups supplemented with oilseed cakes, despite all TM larvae being dried with the same method. LIN 10 larvae showed lower a_w_ compared to CAM 5 (0.45 vs. 0.69; *p* = 0.016), while the other groups displayed intermediate values. Despite the drying process, residual moisture content in the larvae may have influenced their olfactory attributes, potentially leading to the development of less appealing odors. Indeed, some microorganisms can survive even at low a_w_ values, promoting reactions responsible for odorous compound formation.

Other sensory attributes were also affected by diet: color intensity was enhanced in CAM 5 larvae, possibly due to the carotenoid and tocopherol content of camelina cake. However, CAM 10 larvae scored significantly lower. Odor intensity reached its highest value in LIN 10 larvae, whereas LIN 5 obtained the lowest score. Finally, friability was the greatest in the LIN 10 and STD groups, suggesting that both high linseed inclusion and the absence of supplementation may affect larval texture. Interestingly, rancid odor did not differ among groups, indicating that despite higher peroxide value in LIN 10, potential off-flavors associated with lipid oxidation were not perceived. Conversely, fried odor intensity was more pronounced in larvae from oilseed cake diets, especially CAM 5, CAM 10, and LIN 10, which may reflect differences in lipid composition and their role in Maillard reaction and lipid-derived volatiles during cooking. Peanut odor was also particularly high in the LIN 10 and STD groups, which could be linked to the higher presence of linolenic acid in LIN 10 and specific lipid-derived volatiles naturally occurring in STD. In terms of texture, oilseed cake inclusion increased unctuousness of whole dried larvae, consistent with their higher lipid content. Some inconsistencies in these findings may reflect the complex interaction between dietary components and larval metabolism, indicating that the relationship between feed composition and sensory attributes is not linear. Additional studies are therefore needed to clarify these effects and to identify the underlying mechanisms.

## 5. Conclusions

The present study demonstrates that including camelina and linseed cakes in the diet of TM larvae influences key quality attributes relevant for their use as feed and food ingredients. Results indicated that TM larvae can modulate their amino acid contents in response to dietary composition and that moderate inclusion levels (5%) of camelina and linseed cakes may enhance amino acid deposition, whereas higher inclusion levels (10%) may impair protein utilization. Furthermore, camelina and linseed cake inclusion affected specific sensory attributes, indicating that substrate composition can modulate product characteristics beyond nutritional content. Although larvae from the highest linseed inclusion showed increased susceptibility to lipid oxidation during storage, sensory analyses revealed that this did not translate into perceivable rancid off-flavors, and these larvae received the highest overall acceptability scores. These findings suggest that both oilseed by-products (camelina and linseed cakes) can be incorporated at moderate inclusion levels (5%) as sustainable ingredients in TM rearing substrates into TM larval diets to support circular economy objectives without undermining consumer acceptability, provided that inclusion levels are optimized.

## Figures and Tables

**Table 1:** Content of α-tocopherol, δ-tocopherol, γ-tocopherol and β-carotene (mg/kg) in diets, camelina cake and linseed cake included in the diet of *Tenebrio molitor* larvae.

	Cakes		Experimental Diets					
	*Camelina sativa*	*Linum usitatissimum*	STD	CON	CAM 5	CAM 10	LIN 5	LIN 10
α-tocopherol	9.00	3.80	10.6	11.7	8.80	6.40	9.80	8.50
δ-tocopherol	13.0	5.44	0.31	9.05	8.26	9.63	10.8	12.3
γ-tocopherol	149	106	8.00	24.1	26.8	29.4	28.2	35.6
β-carotene	0.41	0.31	0.29	0.26	0.18	0.19	0.22	0.26

STD: diet commonly used by the farm; CON: standard diet; CAM 5: standard diet supplemented with 5% of camelina cake; CAM 10: standard diet supplemented with 10% of camelina cake; LIN 5: standard diet supplemented with 5% of linseed cake; LIN 10: standard diet supplemented with 10% of linseed cake.

**Table 2:** Content of amino acids (mg/100 g as fed) in diets, camelina cake and linseed cake included in the diet of *Tenebrio molitor* larvae.

	Cakes		Experimental Diets					
	*Camelina sativa*	*Linum usitatissimum*	STD	CON	CAM 5	CAM 10	LIN 5	LIN 10
Essential amino acids								
Arginine	1760	1430	750	842	741	732	599	798
Histidine	752	465	492	552	541	537	478	536
Isoleucine	762	500	405	503	470	474	429	490
Leucine	1410	896	879	1003	1033	1083	927	1070
Lysine	1330	875	668	872	778	791	693	807
Phenylalanine	975	705	549	658	624	642	572	657
Threonine	984	580	468	545	528	556	488	545
Valine	1020	603	565	623	600	606	537	605
Non-essential amino acids								
Alanine	1100	740	656	656	656	740	672	740
Aspartic acid	1990	1450	1010	1320	1190	1230	1110	1290
Glutamic acid	4190	3140	254	284	262	260	236	267
Glycine	1210	1010	663	711	688	694	635	677
Proline	1280	620	923	965	1010	1020	933	1010
Serine	1130	861	625	733	687	703	636	717
Tyrosine	500	343	264	323	323	332	293	346
Total	20,349	14,238	11,467	13,160	12,549	12,745	11,386	12,952

STD: diet commonly used by the farm; CON: standard diet; CAM 5: standard diet supplemented with 5% of camelina cake; CAM 10: standard diet supplemented with 10% of camelina cake: LIN 5: standard diet supplemented with 5% of linseed cake; LIN 10: standard diet supplemented with 10% of linseed cake.

**Table 3:** Effect of camelina and linseed cake dietary inclusion on the peroxide value (meq O_2_/kg of fat) of dried *Tenebrio molitor* larvae, evaluated at time 0 (T0) and after 3 months of storage (T3).

	Experimental Groups						RSD ^1^	*p*-Value
	STD	CON	CAM 5	CAM 10	LIN 5	LIN 10		
N. of samples	6	6	6	6	6	6		
Peroxide value								
T0	3.52 ± 0.22	4.07 ± 0.46	4.27 ± 0.53	3.61 ± 0.31	2.95 ± 0.32	2.80 ± 0.88	0.96	0.0791
T3	3.63 ^B^ ± 0.22	2.99 ^B^ ± 0.57	2.43 ^B^ ± 0.65	2.81 ^B^ ± 0.34	2.83 ^B^ ± 0.35	11.3 ^A^ ± 0.88	1.53	<0.0001
RSD	0.55	1.13	1.30	0.77	0.78	2.15		
*p*-value	0.724	0.1783	0.0596	0.1195	0.8029	<0.0001		

^1^ Residual Standard Deviation; STD: diet commonly used by the farm; CON: standard diet; CAM 5: standard diet supplemented with 5% of camelina cake; CAM 10: standard diet supplemented with 10% of camelina cake; LIN 5: standard diet supplemented with 5% of linseed cake; LIN 10: standard diet supplemented with 10% of linseed cake; ^A,B^ values within a row with different superscripts differ significantly at *p* < 0.0001.

**Table 4:** Effect of camelina and linseed cake dietary inclusion on the α-tocopherol, δ-tocopherol, γ-tocopherol and β-carotene contents (mg/kg of larvae) of dried *Tenebrio molitor* larvae, evaluated at time 0 (T0) and after 3 months of storage (T3).

	Experimental Groups						RSD ^1^	*p*-Value
	STD	CON	CAM 5	CAM 10	LIN 5	LIN 10		
N. of samples	6	6	6	6	6	6		
α-tocopherol								
T0	6.32 ^AB^ ± 0.29	3.79 ^D^ ± 0.43	5.45 ^BC^ ± 0.43	7.17 ^A^ ± 0.26	4.63 ^CD^ ± 0.30	5.77 ^B^ ± 0.21	0.61	<0.0001
T3	5.86 ^AB^ ±0.29	4.84 ^BC^ ± 0.53	6.93 ^A^ ± 0.47	6.81 ^AB^ ± 0.26	4.03 ^C^ ± 0.30	5.11 ^ABC^ ± 0.21	0.97	0.0001
RSD	0.72	1.06	1.06	0.65	0.73	0.52		
*p*-value	0.2993	0.1645	0.0465	0.3593	0.1797	0.0541		
δ-tocopherol								
T0	0.23 ^C^ ± 0.003	4.27 ^B^ ± 0.46	4.13 ^B^ ± 0.26	5.27 ^A^ ± 0.27	4.30 ^B^ ± 0.37	5.44 ^A^ ± 0.18	0.47	<0.0001
T3	0.16 ^C^ ± 0.003	3.03 ^B^ ± 0.46	3.70 ^AB^ ± 0.26	4.77 ^A^ ± 0.27	3.31 ^AB^ ± 0.37	4.85 ^A^ ± 0.18	0.91	<0.0001
RSD	0.01	1.12	0.63	0.66	0.91	0.44		
*p*-value	<0.0001	0.0838	0.265	0.222	0.0912	0.0441		
γ-tocopherol								
T0	2.11 ^D^ ± 0.07	4.27 ^C^ ± 0.31	7.21 ^B^ ± 0.55	11.1 ^A^ ± 0.61	6.03 ^B^ ± 0.38	9.82 ^A^ ± 0.30	0.79	<0.0001
T3	1.46 ^E^ ± 0.07	3.54 ^DE^ ± 0.34	7.56 ^B^ ± 0.55^C^	10.6 ^A^ ± 0.61	5.67 ^CD^ ± 0.38	7.87 ^B^ ± 0.30	1.20	<0.0001
RSD	0.16	0.77	1.34	1.50	0.93	0.73		
*p*-value	<0.0001	0.1512	0.6527	0.5652	0.5153	0.0009		
β-carotene								
T0	1.40 ^AB^ ± 0.13	0.81 ^C^ ± 0.06	1.20 ^B^ ± 0.08	1.25 ^B^ ± 0.08	1.13 ^BC^ ± 0.08	1.65 ^A^ ± 0.08	0.20	<0.0001
T3	1.23 ^AB^ ± 0.13	0.83 ^BC^ ± 0.06	1.13 ^ABC^ ± 0.08	1.24 ^AB^ ± 0.08	0.79 ^C^ ± 0.08	1.28 ^A^ ± 0.08	0.23	0.0011
RSD	0.32	0.14	0.19	0.20	0.19	0.19		
*p*-value	0.3949	0.8399	0.5376	0.9335	0.0097	0.0067		

^1^ Residual Standard Deviation; STD: diet commonly used by the farm; CON: standard diet; CAM 5: standard diet supplemented with 5% of camelina cake; CAM 10: standard diet supplemented with 10% of camelina cake; LIN 5: standard diet supplemented with 5% of linseed cake; LIN 10: standard diet supplemented with 10% of linseed cake; ^A–E^ values within a row with different superscripts differ significantly at *p* < 0.0001.

**Table 5:** Effect of camelina and linseed cake dietary inclusion on amino acids content (mg/100 g, fresh weight basis) of 9 weeks old *Tenebrio molitor* larvae.

	Experimental Groups						RSD ^1^	*p*-Value
	STD	CON	CAM 5	CAM 10	LIN 5	LIN 10		
N. of samples	10	10	10	10	10	10		
Essential amino acids								
Arginine	726 ^AB^ ± 16.9	682 ^BC^ ± 15.5	730 ^AB^ ± 16.9	644 ^C^ ± 16.9	755 ^A^ ± 17.8	665 ^BC^ ± 16.9	53.5	<0.0001
Histidine	534 ^AB^ ± 10.6	498 ^B^ ±9.71	567 ^A^ ± 10.6	508 ^B^ ± 10.6	573 ^A^ ± 11.2	537 ^AB^ ± 10.6	33.6	<0.0001
Isoleucine	484 ^C^ ± 10.7	486 ^C^ ± 9.79	532 ^AB^ ± 10.7	483 ^C^ ± 10.7	541 ^A^ ± 11.3	491 ^BC^ ± 10.7	33.9	0.0002
Leucine	944 ^ABC^ ± 17.2	926 ^ABC^ ± 15.7	967 ^AB^ ± 17.2	871 ^C^ ± 17.2	999 ^A^ ±18.2	905 ^BC^ ± 17.2	54.5	<0.0001
Lysine	1064 ^A^ ± 20.4	958 ^B^ ± 18.6	961 ^B^ ± 20.4	858 ^C^ ± 20.4	977 ^AB^ ± 21.5	864 ^C^ ± 20.4	64.4	<0.0001
Phenylalanine	466 ^BC^ ± 8.54	468 ^BC^ ± 7.80	500 ^AB^ ± 8.54	445 ^C^ ± 8.54	510 ^A^ ± 9.00	477 ^ABC^ ± 8.54	27.0	<0.0001
Threonine	511 ^B^ ± 11.3	517 ^B^ ± 10.3	564 ^A^ ± 11.3	510 ^B^ ± 11.3	586 ^A^ ± 11.9	538 ^AB^ ± 11.3	35.8	<0.0001
Valine	680 ^C^ ± 16.5	682 ^C^ ± 15.0	753 ^AB^ ± 16.5	678 ^C^ ± 16.5	778 ^A^ ± 17.4	686 ^BC^ ± 16.5	52.1	<0.0001
Non-essential amino acids								
Alanine	1849 ^A^ ± 128	1845 ^A^ ±117	1631 ^A^ ± 128	1413 ^AB^ ± 128	1908 ^A^ ± 135	891 ^B^ ± 128	406	<0.0001
Aspartic acid	974 ^C^ ± 27.7	1024 ^BC^ ± 25.3	1098 ^AB^ ± 27.7	986 ^BC^ ± 27.7	1174 ^A^ ± 29.2	995 ^BC^ ± 27.7	87.6	<0.0001
Glutamic acid	1563 ^BC^ ± 39.4	1609 ^ABC^ ± 39.4	1671 ^AB^ ± 39.4	1496 ^C^ ± 39.4	1764 ^A^ ± 41.5	1478 ^C^ ± 39.4	124	<0.0001
Glycine	805 ^B^ ± 2.6	735 ^B^ ± 25.2	830 ^AB^ ± 27.6	749 ^B^ ± 27.6	791 ^B^ ± 29.1	933 ^A^ ± 27.6	87.4	<0.0001
Proline	853 ^B^ ± 17.6	827 ^B^ ± 16.0	950 ^A^ ± 17.6	841 ^B^ ± 17.6	957 ^A^ ± 18.5	839 ^B^ ± 17.6	55.6	<0.0001
Serine	693 ^A^ ± 11.2	659 ^AB^ ± 10.2	671 ^A^ ± 11.2	600 ^C^ ± 11.2	688 ^A^ ± 11.8	620 ^BC^ ± 11.2	35.4	<0.0001
Tyrosine	795 ^B^ ± 16.3	791 ^B^ ± 14.9	867 ^A^ ± 16.3	762 ^B^ ±16.3	881 ^A^ ± 17.2	773 ^B^ ± 16.3	51.5	<0.0001
Total	12,942 ^AB^ ± 282	12,707 ^ABC^ ± 258	13,294 ^A^ ± 282	11,846 ^BC^ ± 282	13,881 ^A^ ± 297	11,693 ^C^ ± 285	900	<0.0001

^1^ Residual Standard Deviation; STD: diet commonly used by the farm; CON: standard diet; CAM 5: standard diet supplemented with 5% of camelina cake; CAM 10: standard diet supplemented with 10% of camelina cake; LIN 5: standard diet supplemented with 5% of linseed cake; LIN 10: standard diet supplemented with 10% of linseed cake; ^A–C^ values within a row with different superscripts differ significantly at *p* < 0.0001.

**Table 6:** Effect of camelina and linseed cake dietary inclusion on consumers’ sensory analysis scores of visual, olfactory, and overall acceptance of 9 weeks old *Tenebrio molitor* larvae.

	Experimental Groups						RSD ^1^	*p*-Value
	STD	CON	CAM 5	CAM 10	LIN 5	LIN 10		
N. of consumers	141	141	141	141	141	141		
Visual acceptance	4.79 ^BC^	4.10 ^D^	4.48 ^C^	4.89 ^AB^	4.69 ^BC^	5.05 ^A^	0.15	<0.0001
Olfactory acceptance	5.68 ^A^	3.76 ^E^	4.78 ^D^	5.29 ^BC^	5.10 ^C^	5.65 ^A^	0.14	<0.0001
Overall acceptance	5.29 ^AB^	3.90 ^E^	4.72 ^D^	5.13 ^BC^	4.93 ^CD^	5.42 ^A^	0.13	<0.0001

^1^ Residual Standard Deviation; STD: diet commonly used by the farm; CON: standard diet; CAM 5: standard diet supplemented with 5% of camelina cake; CAM 10: standard diet supplemented with 10% of camelina cake; LIN 5: standard diet supplemented with 5% of linseed cake; LIN 10: standard diet supplemented with 10% of linseed cake; value from 1 to 7 = 1: extremely unacceptable, 2: very unacceptable, 3: moderately unacceptable, 4: neither unacceptable or acceptable, 5: moderately acceptable, 6: very acceptable, 7: extremely acceptable; ^A–E^ values within a row with different superscripts differ significantly at *p* < 0.0001.

**Table 7:** Effect of camelina and linseed cake dietary inclusion on the descriptive sensory analysis scores of 9 weeks old *Tenebrio molitor* larvae.

	Experimental Groups						RSD ^1^	*p*-Value
	STD	CON	CAM 5	CAM 10	LIN 5	LIN 10		
N. of panelists	22	22	22	22	22	22		
Sensory attributes								
Color intensity	61.5 ^C^	81.0 ^AB^	85.9 ^A^	72.2 ^BC^	83.3 ^AB^	78.4 ^AB^	4.99	0.0032
Size uniformity	62.9	57.1	54.7	57.7	54.0	53.7	6.18	0.8158
Odor intensity	82.1 ^B^	77.1 ^BC^	88.2 ^AB^	79.1 ^BC^	67.7 ^C^	97.8 ^A^	5.94	0.0002
Rancid odor intensity	7.18	13.9	12.6	7.50	14.7	10.2	3.87	0.2536
Fried odor intensity	52.9 ^AB^	41.3 ^BC^	56.0 ^A^	55.5 ^A^	39.0 ^C^	60.2 ^A^	6.51	0.0028
Peanut odor intensity	68.0 ^A^	49.0 ^B^	49.0 ^B^	48.1 ^B^	48.4 ^B^	72.9 ^A^	7.68	0.0001
Unctuosity whole	22.1 ^b^	34.4 ^ab^	36.0 ^a^	35.9 ^a^	44.7 ^a^	39.0 ^a^	5.69	0.0255
Unctuosity fragmented	44.4 ^c^	55.1 ^abc^	64.8 ^a^	51.9 ^bc^	59.0 ^ab^	56.0 ^abc^	5.23	0.038
Friability	112 ^A^	59.1 ^C^	53.5 ^C^	90.8 ^B^	52.4 ^C^	109 ^A^	6.74	<0.0001

^1^ Residual Standard Deviation; STD: diet commonly used by the farm; CON: commercial diet; CAM 5: commercial diet supplemented with 5% of camelina cake; CAM 10: commercial diet supplemented with 10% of camelina cake; LIN 5: commercial diet supplemented with 5% of linseed cake; LIN 10: commercial diet supplemented with 10% of linseed cake; values are expressed in mm (1 to 150 mm); ^a,b,c^ values within a row with different superscripts differ significantly at *p* < 0.05; ^A–C^ values within a row with different superscripts differ significantly at *p* < 0.0001.

## Data Availability

Dataset available on request from the authors: the raw data supporting the conclusions of this article will be made available by the authors on request.

## Abbreviations

The following abbreviations are used in this manuscript:

TM	*Tenebrio molitor*
DM	Dry matter
PUFA	Polyunsaturated fatty acids
FA	Fatty acid
FCR	Feed conversion ratio
INEF	Insect Novel Ecologic Food
STD	Standard diet
CON	Control diet
CAM 5	Diet with 5% inclusion of camelina cake
CAM 10	Diet with 10% inclusion of camelina cake
LIN 5	Diet with 5% inclusion of linseed cake
LIN 10	Diet with 10% inclusion of linseed cake
a_w_	Water activity
T0	Time 0
T3	Time 3
FAME	Fatty acid methyl esters
SFA	Saturated fatty acid
MUFA	Monounsaturated fatty acid
EFSA	European Food Safety Authority
